# Vasorelaxant Effect and Blood Pressure Reduction Potential of Pitaya Juice Concentrate (*Stenocereus huastecorum*) Associated with Calcium Channel Blockade

**DOI:** 10.3390/foods13162631

**Published:** 2024-08-22

**Authors:** Yadira Ramírez-Rodríguez, Ricardo Espinosa-Tanguma, Juan Roberto Valle-Aguilera, Aldo A. Rodríguez-Menchaca, Nadia Saderi, Roberto Salgado-Delgado, Elihú Bautista, Luis Garcés, Victoria Ramírez, Karina Robledo-Márquez, Lina Riego-Ruiz, Joyce Trujillo

**Affiliations:** 1 División de Biología Molecular, Instituto Potosino de Investigación Científica y Tecnológica (IPICYT), San Luis Potosí 78216, Mexico; yadira.ramirez@ipicyt.edu.mx (Y.R.-R.); francisco.bautista@ipicyt.edu.mx (E.B.); karina.robledo@ipicyt.edu.mx (K.R.-M.); lina@ipicyt.edu.mx (L.R.-R.); 2 Facultad de Medicina, Departamento de Fisiología y Biofísica, Universidad Autónoma de San Luis Potosí (UASLP), San Luis Potosí 78210, Mexico; espinosr@uaslp.mx (R.E.-T.); vallerob@uaslp.mx (J.R.V.-A.); aldo.rodriguez@uaslp.mx (A.A.R.-M.); 3 Facultad de Ciencias, Universidad Autónoma de San Luis Potosí (UASLP), San Luis Potosí 78210, Mexico; nadia.saderi@uaslp.mx (N.S.); roberto.salgado@uaslp.mx (R.S.-D.); 4 Consejo Nacional de Humanidades, Ciencias y Tecnologías (CONAHCyT), Benito Juárez, Mexico City 03940, Mexico; 5 División de Materiales Avanzados, Instituto Potosino de Investigación Científica y Tecnológica (DMA-IPICYT), San Luis Potosí 78216, Mexico; luis.garces@ipicyt.edu.mx; 6 Departamento de Cirugía Experimental, Instituto Nacional de Ciencias Médicas y Nutrición Salvador Zubirán, Tlalpan, Mexico City 14080, Mexico; victoria.ramirezg@incmnsz.mx

**Keywords:** cactus fruits, *Stenocereus huastecorum*, arterial hypertension, vasorelaxant effect, voltage-gated calcium channels

## Abstract

Arterial hypertension is a highly prevalent chronic disease worldwide, with several etiologies and treatments that may eventually have side effects or result in patients developing tolerance. There is growing interest in traditional medicine and functional foods to isolate biomolecules that could be useful as coadjuvants for treating several aliments. Pitaya, a desert fruit endemic in Mexico, is a rich source of bioactive molecules (betalains and phenolic compounds). In this work, the vasorelaxation properties of pitaya juice concentrate and fraction one were investigated using aortic and mesenteric rings from rats. The incubation of rings with pitaya juice concentrate or fraction one induced significant vasorelaxation, independent of the endothelium, and showed resistance to potassium channel blockers. This vasorelaxation was associated with the transmembrane influx of extracellular calcium through the vascular smooth muscle cells, with an inhibitory effect on the voltage-dependent calcium channel currents. Also, 400 mg/mL of pitaya juice concentrate in spontaneous hypertensive rats reduced their blood pressure for 48 h. Phytochemical analyses showed that the primary compounds in F1 were glycosidic in nature, and could be a complex mixture of disaccharides, dimeric disaccharides, or even tetrasaccharides. The glycosidic compounds found in F1 primarily contributed to vasodilatation, establishing a voltage-dependent calcium channel inhibition as a possible molecular target.

## 1. Introduction

Arterial hypertension (AHT) is a chronic degenerative disease that has continually increased worldwide. In Mexico, it is estimated that more than 30 million people live with AHT, making this the third most expensive disease for public health institutions. Mexico has the highest prevalence of AHT in the world [1,2]. AHT is caused by a persistent and abnormal increase in arterial blood pressure (BP; >130/80 mmHg) in the human. It is determined by cardiac output, blood volume, and systemic vascular resistance [3].

The nervous system regulates BP through chemoreceptor reflexes, which control chemical changes in the blood, and baroreceptor reflexes, which monitor the degree of distension of the vascular walls. The hormonal system regulates BP by altering the cardiac output, adjusting the blood volume, or changing the systemic vascular resistance [4]. These systems are regulated in the blood vessels through smooth muscles and the vascular endothelium [5]. In the vascular endothelium, vasodilator molecules, including nitric oxide (NO), endothelium-derived hyperpolarizing factor, prostaglandins, oxygen radicals, arachidonic acid, and vasoconstrictor agents, such as endothelin, thromboxane A2, angiotensin II, and oxygen radicals, among others, are synthesized and released, and all of them actively participate in BP regulation [5].

In addition, the contractile state of blood vessels depends primarily on the cytosolic calcium (Ca^2+^) concentration, which is determined by the release of Ca^2+^ from intracellular stores and extracellular Ca^2+^ through voltage-dependent Ca^2+^ (Ca_v_) channels. These Ca_v_ channels open in response to depolarization of the membrane potential, which causes muscle contraction, whereas hyperpolarization closes them, inducing relaxation. Therefore, the plasma membrane’s electrical state is fundamental to maintaining the vascular tone [6]. The resting potential of vascular smooth muscle cells (VSMCs) is supported by the activity of the potassium (K^+^) channels, which provide a continuous flow of K^+^ ions into the cells; inhibition depolarizes these cells, opens the Ca_v_ channels, allows Ca^2+^ into the cells, and increases the vascular tone, which generates vasoconstriction. Conversely, hyperpolarization occurs if the K^+^ channels are opened, which closes the Ca_v_ channels and causes relaxation [6,7].

Pharmacological treatments for AHT include various antihypertensive drugs, including angiotensin-converting enzyme inhibitors, angiotensin II receptor blockers, beta-blockers, Ca^2+^ channel blockers, and NO precursors, among others [1]. Despite the wide variety of antihypertensive drugs available, the World Health Organization estimates that approximately two-thirds of patients with AHT in low- and middle-income countries still need access to pharmacological treatments, which is due to the high costs of and difficult adherence to such treatments [8]. There has been growing interest in applying alternative or adjuvant strategies, such as using traditional medicines from medicinal plants or foods rich in bioactive compounds, to control and prevent AHT. In this regard, several studies have focused on describing the vascular protective properties of different bioactive compounds of natural origin, such as the carotenoids in carrot juice [9], flavonoids in musk extract [10], polyphenols in green tea [11], and nitrates in beet juice [12].

“Pitaya” is a columnar cactus fruit endemic in Mexico’s arid and semiarid zones, extending from the southwest U.S. to Venezuela. It includes 24 species, with 80% occurring in various Mexican states. The cactus has pink flowers and edible fruits with various pulp colors and a sweet flavor; it varies in shape and size, is harvested twice a year, and belongs to the genus *Stenocereus* [13]. Previously, the bioactive compounds in the pulp of different pitaya species, namely, *Stenocereus pruinosus*, *Stenocereus stellatus*, *Stenocereus griseus* [14], and *Stenocereus huastecorum* [15], were identified, including ascorbic acid, pectins, sterols, phenolic compounds (quercetin, isorhamnetin, kaempferol, taxifolin, tyrosol, and hydroxycinnamoyl derivatives), flavanones (eridictyol, naringenin), and betalains (indicaxanthin, betanidin, betanin, gomphrenin, phyllocactin, and isophyllocactin), which vary among cactus genera. Several functions have been described including anti-inflammatory, antimicrobial, and antioxidant properties. Also, pitaya has been used to mitigate the effects of non-communicable diseases such as diabetes mellitus and AHT [15].

This study was designed to assess the vasodilatory effects of *S. huastecorum* fruit juice concentrate (PJC) ex vivo and in vivo and to identify the underlying vascular mechanism of action. Moreover, the findings contribute to understanding the vasodilatory properties of PJC and shed light on its potential in therapeutic applications.

## 2. Materials and Methods

### 2.1. Reagents

Phenylephrine (PHE), carbachol, tetraethylammonium (TEA), nifedipine (Nph), propranolol, L-N^G^-nitro arginine methyl ester (L-NAME), terbutaline, DL-dithiothreitol, collagenase type F, papain, and analytical-grade reagents for high-performance liquid chromatography equipped with a diode array detector (HPLC-DAP) analyses were purchased from Sigma-Aldrich (St Louis, MO, USA). A Griess Reagent Kit was purchased from Molecular Probes, Inc. (Eugene, OR, USA), while trifluoroacetic acid was obtained from Karal Analytical Reagents (León, Guanajuato, Mexico). Methyl alcohol (ACS reagent grade) was purchased from CTR SCIENTIFIC (Monterrey, Nuevo León, Mexico). Analytical-grade glacial acetic acid was purchased from Jalmek Científica (San Nicolás de los Garza, Nuevo León, México). All other reagents used were of analytical grade and are commercially available.

### 2.2. Isolation of PJC

Pitaya fruits (*S. huastecorum*) without thorns were obtained during April–May 2019 at the Santa María del Río market, San Luis Potosí (21°48′ N 100°45′ W). Healthy fruits without damage or spots were selected. The pitaya fruits were weighed with and without a shell (5.0 kg vs. 2.7 kg) that was removed manually with a stainless-steel knife. The pitaya juice was extracted with a strainer and a sieve with pore diameters of 1.0 mm to recover the seeds (1.6 L). Finally, the supernatant from the juice was obtained as previously described [15], and this PJC (1.1 L) was used in the study. In total, 550 mL of PJC was lyophilized in 25 mL aliquots to develop ex vivo and in vivo experiments.

### 2.3. Guide for Use and Care of the Experimental Model

All experiments were performed with the approval of the Care Committee and Use of Animals of the Medicine Faculty of the Universidad Autónoma de San Luis Potosí (OFICIO/BGFMUASLP-18-23), according to the National Institutes of Health guide for the care and use of laboratory animals (Publication No. 80-23; revised 1978). The animals were obtained from the Biosciences Center of UASLP. They were maintained under a constant temperature (20–22 °C), humidity (44–66%), and lighting (12 h light/dark cycle) and were fed standard chow and water ad libitum.

### 2.4. Ex Vivo: Measurement of the Vasoactive Response

#### 2.4.1. Experimental Protocol

**Scheme 1:**
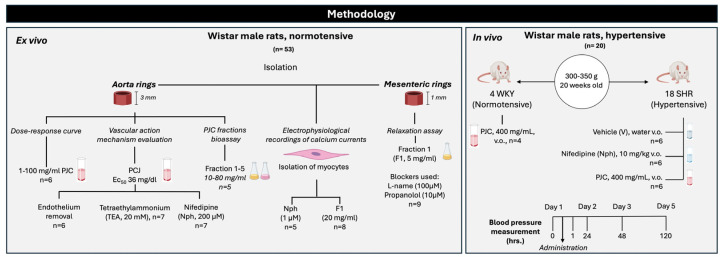
Flowchart showing the experimental design of ex vivo (**left panel**) and in vivo (**right panel**) studies.

Ex vivo experiments were conducted as shown in Scheme 1. To determine the vasoactive effects of PJC, normotensive Wistar rats were used. The aorta (first-order artery) and mesenteric bed (second-order artery) of normotensive male Wistar rats (250–350 g; *n* = 53) were isolated under diethyl ether (1.5 mL) anesthesia, subsequently placed on a dissection plate containing physiological solution (PS; in mM: 135 NaCl, 4.6 KCl, 1.2 KH_2_PO_4_, 1.2 MgSO_4_, 1.1 CaCl_2_, 10 HEPES, and 5 D-glucose, pH 7.4), and the excess connective and perivascular adipose tissues were removed.

Four rings approximately 3 mm long were obtained from each aorta per rat, and each was fixed between two stainless steel hooks in glass chambers for the isolated organ experiments in PS and kept at 37 °C (the different treatments were directly applied to each bath). The upper hooks were connected to an FT.03 tension transducer (Grass Instruments Co, Quincy, MA, USA) connected to a Grass Model 79D Polygraph (Grass Instruments Co, Quincy, MA, USA). Changes in the tension of each ring were registered and analyzed with software developed by the Department of Physiology and Biophysics (UASLP). A passive load of 1 g was applied to each ring and maintained throughout a 30-min equilibration period, followed by a pre-condition period consisting of 50 min (including incubations of 15 min PHE, 5 min carbachol an endothelium-dependent vasorelaxant, 15 min wash, and 15 min PHE), after which, the (PJC or F1) was proven.

To test the relaxation effect of PJC, a dose–response curve was initially generated with lyophilized PJC (1, 5, 10, 25, 75, and 100 mg/mL) for the aortic rings precontracted with PHE (10 μM). Endothelium elimination was determined by the absence of relaxation upon carbachol (10 μM, 5 min) action. Additionally, the absence of endothelium was indirectly verified by the production of NO through the concentration of nitrites in the medium of the isolated aorta rings, using the Griess reaction and a standard curve with a commercial kit. The concentration of nitrites in the medium of the rings with endothelium was (0.53 ± 0.11 µM NO_2_) and without endothelium was (−3.5 ± 0.39 µM NO_2_), confirming the lack of endothelium NO production, and finally, by histology (hematoxylin–eosin; Supplementary Figure S1). Then, the rings were washed with PS and contracted again with PHE (10 μM). After the contraction stabilized, 50% of the rings received increasing concentrations of PJC individually, while the other half received PS as a control. The relaxation effect was recorded for 30 min. The half-maximal effective concentration (EC_50_) was calculated with the dose–response curve and used for subsequent assays. Six rats were used for this experiment, and four aortic rings per rat.

Once the EC_50_ was established, the mechanism responsible for the vasorelaxant effect associated with PJC was evaluated. Experiments were conducted with aortic rings precontracted with PHE (10 μM) from normotensive male Wistar rats. These experiments were carried out by (1) endothelium withdrawal using 5% carbogen (2 min); (2) nonselective K^+^ channel blocker preincubation (TEA; 20 mM) for 10 min; and (3) with a Ca_v_ channel blocker (Nph; 200 nM). Control experiments were conducted in the absence of antagonists for each experiment. Additionally, the smooth muscle integrity was assessed by contraction with PHE (10 μM) after the treatments. At least five rats were used for each experimental group, and four aortic rings were used per rat.

In addition, a bioassay was performed with eight fractions, F1–F8, obtained by reverse-phase chromatography to identify the fraction responsible for the vasorelaxant effect of PJC, but the samples of F6, F7, and F8 were insufficient for evaluation. Samples F1–F5 were evaluated in aortic rings precontracted with PHE (10 μM) and without endothelium, and four concentrations (10, 20, 40, and 80 mg/mL) were evaluated from each of the fractions (F1–F5). Additionally, the highest concentration (80 mg/mL) of F1 was observed to damage the integrity of the rings, preventing them from contracting again when PHE (10 μM) was added. Therefore, this concentration was not considered when establishing a mean dose for the patch clamp analysis (20 mg/mL).

The second-order arteries (resistance vessels) measured approximately 350 µm in diameter, and 1 mm long segments of the mesenteric artery were obtained and individually mounted for isometric recording with an AE801 micro-transducer [16] (Sensor One Technologies, Oakland, CA, USA) and two copper microwires (40 µm in diameter and ~1 cm in length). These microwires passed through the lumen. One was fixed to a micrometer for length adjustments, and the other was connected to a force transducer. Isometric tension changes were analyzed with a LINSEIS L6512B recorder (Robbinsville, NJ, USA). Once the mesenteric rings were mounted (PS, at 37 °C), a passive load of 8–10 mg was applied for 10 min.

Of all the fractions obtained from the PJC, only F1 exhibited significant relaxation, and it was the only fraction used during further tension experiments. The dose used for F1 (5 mg/mL) during the experiments was obtained from the dose–response curves prepared previously. The endothelium was removed by inserting a 70 μm diameter silk thread into the arterial lumen and creating frictional motions against the wall. Propranolol, a β-adrenergic receptor blocker (10 µM, 15 min), and L-NAME, an endothelial NO synthase blocker (100 µM, 5 min), were present in the PSs of all the experiments. Endothelial functionality was confirmed by the lack of relaxation when carbachol (10 µM, 5 min) was added during the plateau of the PHE-induced contraction (100 µM). In addition to the β-adrenergic receptor blockade, relaxation was not induced by adding terbutaline (1 µM, 5 min), a β-adrenergic agonist. The smooth muscle integrity was evaluated by contraction with PHE (100 µM), and control experiments were conducted for each experiment by adding PS. Nine rats were used for these experiments.

#### 2.4.2. Isolation of Smooth Muscle Cells from the Thoracic Aorta and Mesenteric Artery for Patch-Clamp Recording

The thoracic aorta and mesenteric artery were stripped of adipose and connective tissue and soaked separately in a cell dissociation solution (CDS; mM: 55 NaCl, 6 KCl, 80 C_5_H_9_NO_4_, 80 NaOH, 2 MgCl_2_, 10 C_6_H_12_O_6_, and 10 HEPES). Subsequently, the tissues were sectioned into strips, placed in a tube with 700 µL of CDS, along with DL-Dithiothreitol (5 mM), papain (1.4 mg/mL), and bovine serum albumin (2 mg/mL), and preincubated at 37 °C for 10 min. The cells underwent enzymatic digestion at 120 rpm and 37 °C for 32 min (thoracic aorta) and 12 min (mesenteric artery). Next, the tissue strips were transferred to another tube with 700 µL of CDS, 1.4 mg/mL of type F collagenase, and 100 mM calcium chloride. This mixture was incubated at 37 °C for 12 min (thoracic aorta) and 6 min (mesenteric artery). Finally, the tissue was submerged in an enzyme-free CDS and the cells were dissociated with a 1 mL micropipette tip.

#### 2.4.3. Electrophysiological Recordings

VSMCs were obtained from both arteries, placed directly at the bottom of the experimental chamber, and observed with an inverted microscope (Zeiss Axio Vert.A.1). For 10 min before the experiments, the cells were allowed to adhere to the glass bottom of the chamber. The cells were recorded in whole-cell mode with micropipettes prepared with borosilicate glass capillaries (WPI, Sarasota, FL, USA). After filling them with the internal solution, the pipettes had 2–3 mΩ resistance. The ionic currents were recorded with an Axopatch 200B amplifier (Molecular Devices, Sunnyvale, CA, USA). The acquisition and generation of the voltage pulse protocols were conducted with the Digidata 1440A interface and pCLAMP 10 software (Molecular Devices). The currents were filtered, subjected to leak subtraction (P/4) through the amplifier, and digitized, and the files were stored for subsequent analysis.

To isolate the currents mediated by the Ca^2+^ channels, the pipettes were filled with a high-cesium-ion solution (in mM: 132.5 cesium chloride, ATP disodium salt hydrate, 0.1 guanosine triphosphate, 3 MgCl_2_, 10 EGTA, and 10 HEPES) at pH 7.2. Barium (Ba^2+^) ions were used to record the currents passing through the Ca^2+^ channels. The solution based on Ba^2+^ consisted of (mM) 10 BaCl_2_, 150 NaCl, 5.4 C_6_H_12_O_6_, and 5 HEPES, pH 7.4. The mean concentration of F1 (20 mg/mL) was used for these experiments, and Nph (1 µM) served as the control. Nine rats were used for each experimental group.

### 2.5. In Vivo Studies: Measurement of the Vasoactive Response

#### 2.5.1. Experimental Protocols

In vivo experiments were conducted as shown in Scheme 1. To confirm the hypotensive effect of PJC, we decided to prove the ex vivo effects in a biological model with spontaneously hypertensive rats (SHRs). Twelve SHRs were used to establish the dose of PJC necessary to reduce the BP levels by ~20%. Moreover, eighteen SHRs and four Wistar Kyoto (WKY, control) rats were included to evaluate the antihypertensive effect of PJC. Both rat sets were 20 weeks old (300–400 g), obtained from the Universidad Nacional Autónoma de México (UNAM), and used for the experiments. The animals were kept at a constant temperature (18–22 °C) and under constant lighting (12 h light/dark cycle), fed with standard food and water ad libitum, and acclimatized for two weeks.

#### 2.5.2. PJC Effect in SHR and WKY Rats

The PJC dose was determined beforehand with twelve rats (pilot group; *n* = 3) that were administered escalating doses of PJC (0, 200, 400, and 600 mg/mL), and after completing PJC administration, BP was registered at 0, 1, 24, and 48 h. Eighteen SHRs (20 weeks old, 300–400 g) were randomly divided into three groups: the vehicle group (V, water, *n* = 5), who received 1 mL of water orally (p.o.); the second group, who received 10 mg/kg of nifedipine (p.o.) dissolved in ethanol, used as a positive control (Nph, *n* = 6); and finally, the third group, who received 400 mg/mL of PJC (p.o.) dissolved in water (PJC, *n* = 6). A group of normotensive WKY rats + PJC (WKY + PJC, *n* = 4) was included. The treatment consisted of a single initial dose administered for 5 days.

The systolic and diastolic BP were measured for all studied groups every 24 h for two weeks with the CODA tail-cuff BP measurement system (Kent Scientific, Torrington, CT, USA). Once the rats reached stable high BP levels, they were administered V, Nph, and PJC, as described for the three groups. The BP was measured at 0 min and at 1, 24, 48, and 120 h.

### 2.6. Phytochemical Analysis

#### 2.6.1. PJC Fractionation

PJC fractionation was performed chromatographically with a C-18 reverse-phase column (8 cm h × 8 cm i.d., frs. 500 mL), eluted by gravity (250 g of Chromabond^®^ Sorbent C18 silica gel). A total of 20 g of lyophilized PJC was dissolved in 20 mL of 1% acetic acid (CH_3_COOH), and elution was performed with solvent mixtures of 1% CH_3_COOH, 1% CH_3_COOH-methanol (MeOH) 98:2, 1% CH_3_COOH-MeOH 95:5, 1% CH_3_COOH-MeOH 90:10, 1% CH_3_COOH-MeOH 80:20, 1% CH_3_COOH-MeOH 70:30, 1% CH_3_COOH-MeOH 50:50, and 100% MeOH to obtain eight fractions separated by polarity. Fractions with less than 5% MeOH were recovered by lyophilization, while fractions with >5% MeOH underwent vacuum distillation in a rotary evaporator (100 mBar, 40 min at 50 °C), followed by lyophilization. The fractions were stored at −20 °C and protected from light until further use. They were subsequently subjected to ex vivo bioassays, as described below, to identify the vasoactive fraction and, subsequently, profile it for compound identification.

#### 2.6.2. Bioactive Compounds Profiling by High-Performance Liquid Chromatography Equipped with a Diode Array Detector (HPLC-PDA)

Independent 250 mg samples of lyophilized PJC and the vasoactive F1 were dissolved in 10 mL of an aqueous 0.1% trifluoroacetic acid–MeOH 80:20 mixture. The resulting mixture was centrifuged at 4500 rpm for 10 min at 4 °C. The supernatant was recovered and filtered through a polypropylene membrane (0.45 µm) for analysis by HPLC-PDA.

HPLC-PDA equipment (1200 Agilent chromatograph-Agilent G1315D) was used. Separations were carried out with a Purospher^®^ STAR RP 5 µm column (250 × 4.6 mm, Merck-Sigma, St Louis, MO, USA) and gradient elution; solvent A was 0.1% trifluoroacetic acid and solvent B was MeOH. The optimized elution gradient to separate the compounds was as follows: 0% B, 0 min; 5% B, 15 min; 15% B, 25 min; 100% B, 30–35 min; and 0% B, 40–45 min. A 0.4 mL/min flow rate was used, and the detection wavelengths were 254 nm for phenolic compounds and 480 nm for betalains, with an injection volume of 50 µL.

#### 2.6.3. Characterization by UV-Vis Spectroscopy

The spectra of the samples were measured in the range from 200 to 800 nm with a Perkin-Elmer Lambda 900 UV-Vis-NIR spectrometer. Four milligrams of PJC and F1 were dissolved in 1 mL of deionized water and placed in 1 cm polypropylene cells, and the peak absorbance and wavelength were determined.

#### 2.6.4. NMR Analysis of PJC and F1

A 20 mg PJC or F1 sample was dissolved in 0.75 mL of a mixture of D_2_O-TFA-*d* 99.5:0.5 using 0.5 mm NMR tubes and analyzed on a Bruker Avance III HD 700 MHz. The raw data were processed using the MestReNova software version 15.0.1 (Mestrelab Research; Coruña, España), and the scale was referenced using the residual peak of D_2_O (4.79 ppm) for the ^1^H NMR spectrum and the residual peak of TFA-*d* (164.20 ppm) for ^13^C.

### 2.7. Statistical Analyses

All data are expressed as means ± SEM. Normality was assessed with the Shapiro-Wilk test and statistical significance between groups was evaluated with one-way ANOVA. For differences between groups, the Tukey-Kramer test was employed. Values were considered significant at *p* < 0.05. The data were analyzed with GraphPad Prism 5 software (GraphPad Software, La Jolla, CA, USA).

## 3. Results

### 3.1. PJC-Induced Relaxation of Isolated Thoracic Aortic Rings

**Figure 1:**
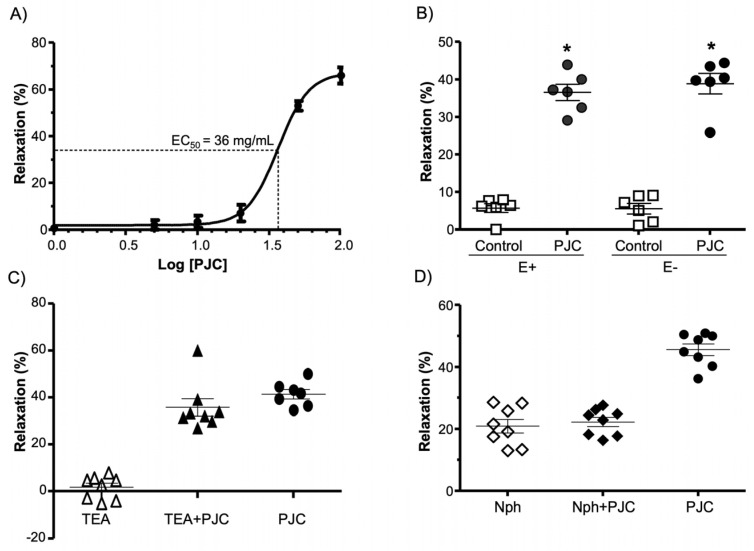
Analysis of the pitaya juice concentrate (PJC) vasoactivity. (**A**) Log dose–response curve (1, 5, 10, 25, 75, and 100 mg/mL) cumulative type (*n* = 6). The half-maximal effective concentration (EC_50_) was obtained by fitting the individual log dose–response curves of PJC. (**B**) PJC effect (36 mg/mL; *n* = 6; -dark circles-) on rings with endothelium (E +), without endothelium (E-), and the control group (-white squares-; physiological solution; PS). (**C**) PJC effect (36 mg/mL; *n* = 7) on rings in the presence (-dark triangles) and absence (-dark circles-) of tetraethylammonium (TEA, 20 mM), a potassium channel blocker, and -white triangles- show control TEA group. (**D**) PJC effect (36 mg/mL) on rings in the presence (-black diamonds-) and absence (-black circles-) of nifedipine (Nph, 200 nM), a voltage-dependent calcium channel blocker, and -white diamonds- show control Nph group. Relaxation was calculated as a percentage of the initial contraction. All analyses were on thoracic aortic rings; data are expressed as means ± SEM. * *p* < 0.05 vs. control.

Figure 1 shows the effect of vasoactive PJC. Thoracic aortic rings were isolated from normotensive male Wistar rats to establish the EC_50_ of the PJC vasodilatation effect. Panel A shows a dose–response curve for PJC (1–100 mg/mL, *n* = 6). It was observed that PJC induced a dose-dependent relaxation of the aortic rings with endothelium that were previously contracted with PHE (10 μM). The maximum relaxation rate was 66 ± 3.4% for the 100 mg/mL dose. The EC_50_ was 36 ± 2.9 mg/mL, with a 34 ± 2% relaxation rate. The EC_50_ (36 mg/mL of PJC) was used in the subsequent experiments.

### 3.2. Role of the Endothelium in the Vascular Response to PJC

Similarly, to identify the role of the endothelium in the vasodilator response of PJC, aortic rings were incubated in two conditions. The first experiment showed that PJC incubation (Figure 1B, -black circles-) exerted a comparable relaxation effect on thoracic aortic rings in the presence of endothelium (E+) and the absence of endothelium (E−), with values of 38 ± 6% and 40 ± 5%, respectively, compared to the control (PS, Figure 1B -white squares-). The rings were previously contracted with 10 µM PHE. The vasodilator effect induced by PJC was endothelium-independent.

### 3.3. Role of K^+^ Channel in the Vascular Response to PJC

Further, to identify a possible target for PJC, we probed the K^+^ channels’ activation in the vasodilatation caused by PJC. The tension of the isolated thoracic aorta rings precontracted with 10 µM PHE was evaluated in the presence and absence of 20 mM TEA, a nonselective K^+^ channel blocker. Figure 1C shows the relaxation percentages exhibited by the rings treated only with TEA (2 ± 2%; -white triangles-), TEA + PJC (37 ± 3%; -black triangles-), and those treated only with PJC (39 ± 2%; black circles). These results showed no significant difference between the rings treated with TEA + PJC and those treated exclusively with PJC, suggesting that PJC produced vasodilation, but not through the activation of the K^+^ channels.

### 3.4. Role of Ca_v_ Channels in the Vascular Response to PJC

In additional experiments, the participation of Ca_v_ channels was studied by inhibiting their activity with a selective blocker, Nph, in isolated thoracic aorta rings. Figure 1D shows the relaxation percentages obtained with Nph (200 nM; -white diamonds-), Nph + PJC (-black diamonds-), and PJC alone (-black circles-). The rings that received Nph presented a relaxation rate of 21 ± 2%; similar results were obtained with the Nph + PJC treatment (24 ± 2%), while the rings exposed exclusively to PJC presented 46 ± 2% vasodilatation, indicating that the effect of PJC was inhibited by blocking the Ca_v_ channels with Nph. Therefore, the relaxation effect of PJC was partly due to the blocking of the Ca_v_ channels.

### 3.5. PJC Fractions Relaxed Isolated Thoracic Aorta and Mesenteric Artery Rings

**Figure 2:**
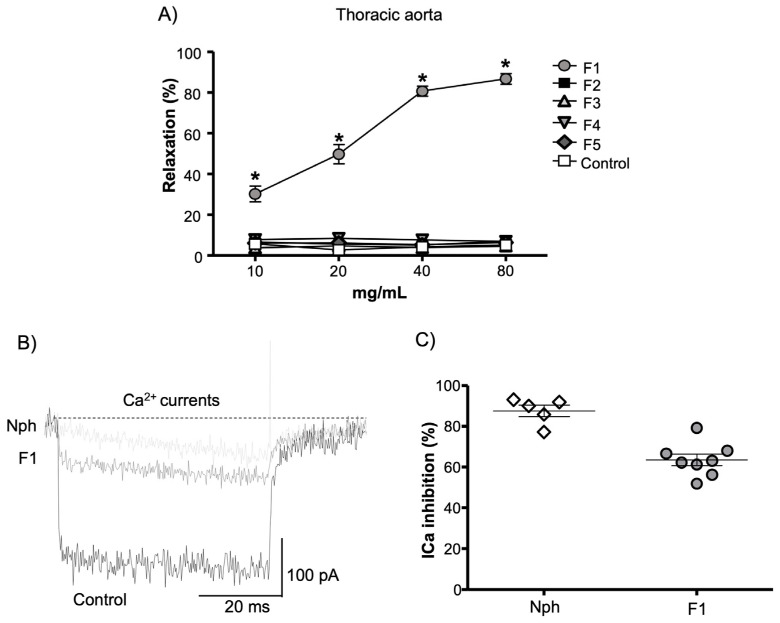
Vasoactivity analyses of pitaya juice concentrate (PJC) fractions. (**A**) Percentage relaxation at different concentrations (10, 20, 40, and 80 mg/mL; *n* = 5) of PJC fractions 1–5 (F1–F5) for isolated thoracic aorta rings precontracted with phenylephrine (PHE, 10 µM) and white squares show control group. (**B**) Representative traces of barium (Ba^2+^) currents through voltage-dependent calcium channels in thoracic aorta myocytes were recorded at +20 mV from a holding potential of −90 mV in basal state (control, black trace), fraction 1 (F1; 20 mg/mL, *n* = 8; dark gray trace), and nifedipine (Nph; 1 μM, *n* = 5; light gray trace). (**C**) Percentage inhibition of calcium (Ca^2+^) currents with Nph (1 μM; -white diamonds-) and F1 (20 mg/mL; gray circles). Relaxation was calculated as a percentage of the initial contraction. Data are expressed as means ± SEM. * *p* < 0.05 vs. control. Milliseconds (ms), Millivolts (mV), and picoAmperes (pA).

A bio-directed polarity assay was used to identify the targeted compounds associated with the vasorelaxant effect, as previously described. As Figure 2A shows, the F1–F5 fractions obtained from PJC were individually evaluated to identify which caused a vasoactive effect in the conductance artery. Of the five fractions evaluated, only F1 exhibited a vasoactive effect, causing a dose-dependent relaxation of the aortic rings. The maximum relaxation rate was 81 ± 3.4% at a concentration of 40 mg/mL. In contrast, fractions F2–F5 do not show a vasoactive effect, like the control group (PS). Similar results were observed when evaluating the relaxation effect of F1 in resistance arteries, specifically in isolated mesenteric artery rings (Supplementary Figure S2A). F1 (5 mg/mL; -gray circles-) produced 31 ± 2% relaxation of the mesenteric rings compared with only 4 ± 1% for the control group (PS; -white squares-). F1 from PJC was the only fraction that demonstrated a vasorelaxant effect on conduit and resistance arteries.

### 3.6. Ca_v_ Channels in the Vascular Response To F1 from PJC

Subsequently, the effect of F1 on the Ba^2+^ currents through Ca_v_ were evaluated by patch-clamp experiments on freshly isolated myocytes from the thoracic aorta and mesenteric arteries. Figure 2B (aorta) and Supplementary Figure S2B (mesenteric) show representative traces for Ba^2+^ currents in the basal state (control; -black trace-) with the presence of 20 mg/mL of F1 (dose previously established; -black-gray trace-) or 1 μM of Nph (-light-gray trace-). Figure 2C shows the percentage inhibition of calcium currents, where 88 ± 3% Ba^2+^ current inhibition through the Ca_v_ channels was observed when Nph was added to the aortic rings (-white diamonds-). Notably, for F1, the myocyte currents exhibited an inhibition of 65 ± 3% (-gray circles-).

For the mesenteric arteries, currents from five isolated myocytes were registered, which showed an average inhibition of 63 ± 8% (Supplementary Figure S2C, -gray circles-). These findings decisively demonstrate that F1 exerted a significant inhibitory effect on Ca_v_ channels and support the hypothesis that these channels play a pivotal role in the PJC-induced vasodilator response.

### 3.7. PJC Induces Antihypertensive Effects in SHRs

Once the effects of PJC and F1 were established ex vivo in normotensive rats, the response to PJC was evaluated using an in vivo model in AHT conditions, including in SHRs, which are genetically hypertensive rats [17].

First, a pilot study was conducted to establish the dose required to reduce BP by 20% in the SHRs. Supplementary Figure S3 shows the systolic (A) and diastolic (B) BP, respectively, in the SHRs. The SHRs received PS as a vehicle (*n* = 3; -white squares-) and showed a high systolic and diastolic BP for 48 h, at 147 ± 6 mmHg and 105 ± 5 mmHg, respectively. The animals that were administered a single initial dose of PJC of 200 (*n* = 3; -black circles-), 400 (*n* = 3; -black-gray circles-), or 600 mg/mL (*n* = 3; -light gray circles-) showed reductions in their systolic and diastolic BP over 48 h (25 ± 5% and 29 ± 1%; 25 ± 3% and 28 ± 1%; and 32 ± 2% and 28 ± 1%, respectively). It should be noted that the group that received 400 mg/mL of PJC exhibited a significant hypotensive effect after the first hour of administration, and this effect was sustained until 48 h. Therefore, this dose was used for subsequent experiments.

**Figure 3:**
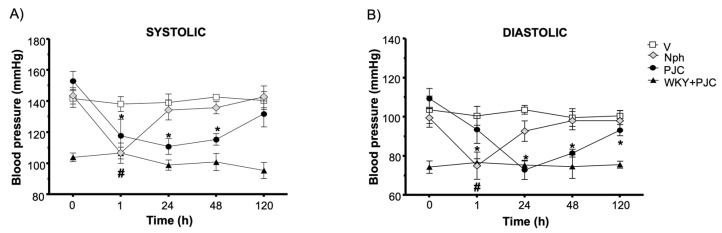
Administration of pitaya juice concentrate (PJC) to spontaneously hypertensive rats (SHRs). (**A**) Systolic blood pressures at 0, 1, 24, 48, and 120 h and (**B**) diastolic blood pressures at 0, 1, 24, 48, and 120 h. Blood pressure recorded (mmHg) in SHRs and Wistar Kyoto (WKY) rats. The SHRs were administered the vehicle (V; water, *n* = 5), PJC (400 mg/mL, *n* = 6), and nifedipine (Nph; 10 mg/kg, *n* = 6), and the WKY rats received only PJC (400 mg/mL, *n* = 4). Data are expressed as means ± SEM. # *p* < 0.05 vs. Nph 0 h, * *p* < 0.05 vs. CJP 0 h, and *p* < 0.05 vs. V.

Consequently, as shown in Figure 3, the BP was recorded for all animals over five consecutive days. The group receiving V (water, -white squares-) showed elevated BP throughout the trial (systolic BP of 139 ± 1 mmHg (panel A) and diastolic BP of 102 ± 1 mmHg (panel B)), while the group that received 10 mg/mL of Nph (-gray diamonds-) exhibited a 21 ± 3% decrease in systolic BP and a 24 ± 3% decrease in diastolic BP. However, one hour after drug administration, the values of systolic BP were 107 ± 6 mmHg and diastolic BP were 75 ± 6 mmHg; however, this effect disappeared after 24 h. Interestingly, the SHRs that received PJC orally had their systolic BP reduced by 20 ± 2% (118 ± 10.5 mmHg) and diastolic BP reduced by 19 ± 3% (85 ± 10.1 mmHg) within 60 min after administration. Remarkably, these reductions were maintained for 48 h by the systolic (~25%; 120 ± 5.7 mmHg) and diastolic BP (~22%; 85 ± 3.6 mmHg), an effect that did not persist at 120 h. As expected, the WKY animals administered with PJC showed normotensive BP levels (systolic BP of 107 ± 2 mmHg and diastolic BP of 77 ± 2 mmHg). This suggested that PJC exerted a sustained antihypertensive effect at 0, 24, and 48 h in the in vivo model.

### 3.8. PJC and F1 Profiling

**Figure 4:**
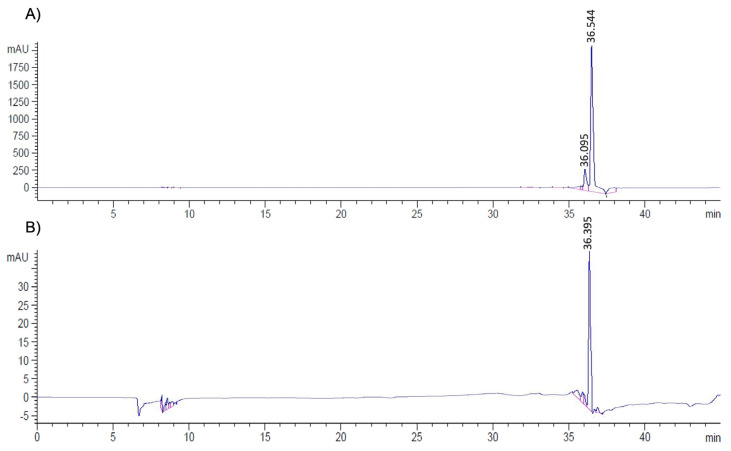
High-performance liquid chromatography equipped with a diode array detector (HPLC-PDA) profiles at 480 nm for (**A**) pitaya juice concentrate and (**B**) fraction 1 (CH_3_COOH 1%). Milli-Absorbance Units (mAU).

**Figure 5:**
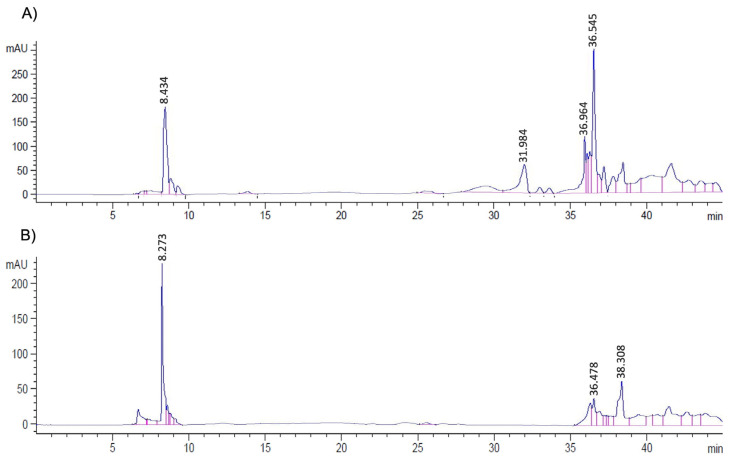
High-performance liquid chromatography equipped with a diode array detector (HPLC-PDA) profiles at 254 nm for (**A**) pitaya juice concentrate and (**B**) fraction 1 (CH_3_COOH 1%). Milli-Absorbance Units (mAU).

The next step was to profile the PJC and F1 with HPLC-PDA. As shown in Figure 4, the metabolite profiling of PJC (A) and F1 (B) was performed with HPLC-PDA at 530 and 480 nm to detect the betalains, and at 254 nm to detect the phenolics and flavonoids, as shown in Figure 5A,B. The profiling results indicated that, at 480 nm, the chromatograms for PJC vs. F1 showed only one prominent peak with a retention time of 36.39 min. However, the absorbance of the peak observed for F1 was 45 times lower than that for the same peak in PJC, suggesting that the concentration of betalains in F1 decreased significantly. The HPLC-PDA profiles at 254 nm showed losses in the peaks with retention times of 29.5, 31.9, and 36.6 min in the F1 chromatogram from PJC. Decreases in the absorbance values of the peaks with retention times between 36.0–36.5 and 41.4 min also suggested decreases in the number of compounds with aromatic residues, such as phenolics and flavonoids, as product of the chromatographic separation process.

### 3.9. Characterization of PJC and F1 by UV-Vis Spectroscopy

**Figure 6:**
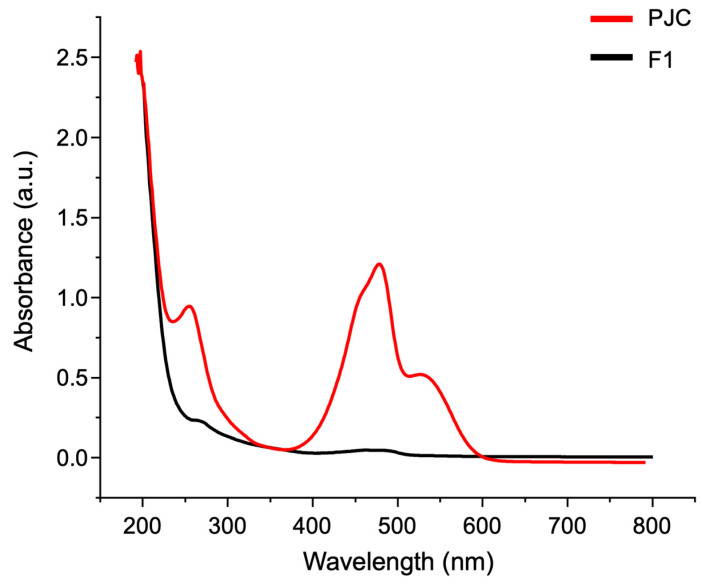
UV-Vis absorption spectra of the pitaya juice concentrate (PJC) and fraction 1 (F1, 1% CH_3_COOH).

UV–Vis spectroscopic data were obtained to corroborate the inferences made from the HPLC-PDA analyses. The UV-Vis spectrum of PJC (-red line-), as shown in Figure 6, exhibited two absorption peaks near 485 and 530 nm, characteristic of betalains. In contrast, the UV-Vis spectrum of F1 (-black line-) showed no peaks with absorbances between 485 and 530 nm; only a weak peak near 280 nm was observed. However, due to the high concentration of the sample (4 mg/mL), it can be inferred that the concentrations of phenolic and/or flavonoid compounds in F1 were very low.

### 3.10. NMR Analysis of F1

The ^1^H NMR spectrum of F1 displayed two signals with chemical shifts and multiplicities distinctive for anomeric protons at *δ*_H_ 5.00 (d, *J* = 3.8 Hz) and 4.41 (d, *J* = 8.0 Hz), indicating a glycosidic nature (Supplementary Figure S4). In the same spectra, a set of signals associated with these sugar moieties in the range of *δ*_H_ 3.02–3.88 was observed. In the ^13^C NMR spectrum, three sets of signals were displayed. The first set of signals were at *δ*_C_ 172.14–178.60, the second at *δ*_C_ 93.42–103.70, and the third at *δ*_C_ 61.62–82.69 (Supplementary Figure S5). The signals shown in the ^13^C NMR spectra supported the hypothesis of compounds of a saccharide nature in F1.

## 4. Discussion

In previous studies using ultra-performance liquid chromatography-tandem mass spectrometry (UPLC-PDA-ESIMS), it was described that fruits such as the pitaya (*S. huastecorum*) contain several different bio-compounds. Among those that stand out is indicaxanthin, a betaxanthin known for its vasodilator activity [15,18]. However, there is no clear scientific evidence associated with the antihypertensive effect of pitaya. Nevertheless, many plants are reported to control and prevent AHT [19]. Other cactus fruits such as prickly pear (*Opuntia ficus*-*indica*) [20]; flowers such as roselle (*Hibiscus sabdariffa*) [21]; and roots such as beet (*Beta vulgaris* L.) [22] have been associated with antihypertensive activity. This effect is related to the high contents of betalains, anthocyanins, and phenolic compounds. Our group previously reported increased NO metabolites after PJC administration in the cisplatin-nephrotoxic model [15]. Therefore, there is an interest in assessing the vasodilatory effects of fruit PJC (*S. huastecorum*) and identifying the underlying vascular mechanism of action. Therefore, we performed several experiments in which conductance and resistance vessels were included. Conductance vessels, like the thoracic aorta, conduct oxygenated blood in the body. Resistance vessels include small arteries, arterioles, and precapillary sphincters responsible for regulating blood flow and BP. Specifically, in walls, there are VSMCs that control the vascular tone by generating peripheral resistance to blood flow and determining the BP [5]. This study evaluated the effect of PJC on both types of vessels. Firstly, it was observed that PJC-induced relaxation dose-dependent in aortic rings with endothelium. An EC_50_ of 36 ± 2.9 mg/mL was established, with a 34 ± 2% relaxation rate. The vascular endothelium is an essential regulator of BP and is linked to the local release of vasoconstriction factors (endothelin) and vasorelaxation factors (prostacyclin and NO) [23]. For this reason, identifying the role of the endothelium in the vasodilator response of PJC is crucial. It was found that the vasoactive effect was similar with or without endothelium (38 ± 6% and 40 ± 5% relaxation, respectively), indicating that it is endothelium-independent. In this regard, Sarr et al. (2009) observed the opposite effect. They analyzed the relaxation effect of an extract from *H. sabdariffa* on isolated thoracic aorta rings, establishing that 10^−4^–10^−1^ mg/mL of *H. sabdariffa* extract produced dose-dependent relaxation in the presence of endothelium and rings previously contracted with 1 µM norepinephrine, showing a maximum relaxation of 66 ± 8%. When the endothelium was removed, the relaxation effect decreased significantly (24 ± 0.5%), indicating that this relaxation effect was endothelium-dependent and related to the phenolic acid and anthocyanin contents by phytochemical analysis [21]. In this study, the vasodilator effect may have varied due to differences in the bioactive compounds present in PJC compared to those in *H. sabdariffa*, which will be detailed later.

The activation of K^+^ channels in PJC induced vasodilation in isolated thoracic aorta rings precontracted with PHE was evaluated by blocking non-selective K^+^ channels with TEA. The relaxation percentage was very similar when administering TEA + PJC or only PJC (37 ± 3% vs. 39 ± 2%), suggesting that PJC produced vasodilation by different mechanisms. This contrasts with previous studies, suggesting that relaxation by *Valeriana wallichii* [24] and *H. sabdariffa* [21] was mediated by K^+^ channels because the resting potentials of the VSMCs were maintained with these channels, which remained open at the usual potentials of the VSMCs and enabled the continuous efflux of K^+^ from the cell. Opening more K^+^ channels hyperpolarized the cell membrane, inhibited Ca^2+^ entry through the Ca_v_ channels, and, thus, caused vascular relaxation [6,18].

Another possible mechanism involves the Ca_v_ channels, because they are essential targets in treating AHT. Designing antihypertensive therapies to modulate Ca^2+^ entry into cells is crucial in this context. This study evaluated the participation of Ca_v_ channels using a selective blocker called Nph in isolated thoracic aorta rings. The rings that received Nph presented the expected relaxation (21 ± 2%) [25], with a similar value when Nph + PJC treatment was applied (24 ± 2%). Only PJC induced a relaxation of 46 ± 2%, indicating that this effect was partly due to blocking the Ca_v_ channels. It is known that Ca^2+^ activates contractile proteins, and in VSMCs, this ion is introduced by Ca_v_ channels during depolarization, causing smooth muscle contraction [6,26]. In AHT, the overexpression of Ca_v_ channels causes increased Ca^2+^ influx into VSMCs, driving the development of an abnormal vascular tone and elevating peripheral vascular resistance [27].

Furthermore, to identify the targeted compounds associated with the vasorelaxant effect, a bio-directed study was carried out with PJC. Five fractions were separated by polarity and individually evaluated to identify which had a vasoactive effect on conductance and resistance vessels. The maximum relaxation rate was obtained only by F1 in both conduction vessels (~70% with EC_50_; aorta artery) and resistance vessels (~31% with 5 mg/mL; mesenteric artery). This can be associated with significant differences in vascular reactivity occurring as a function of the concentrations/doses of vasoactive compounds (for example, bioactive compounds), vessel size, and anatomical location [28,29,30]. Once it was established that the fraction of PJC was responsible for the vasorelaxant effect and was partially mediated through Ca^2+^ channels, the next step was to determine if these Ca_v_ channels were active and if F1 from PJC modulated them in patch-clamp experiments with myocytes isolated from conductance vessels and resistance vessels. It was observed that, as expected, Nph inactivated Ba^2+^ currents through Ca_v_.

Interestingly, F1 from PJC inhibited these currents in both the aorta and mesenteric arteries with similar percentages (65 ± 3% and 63 ± 8%), suggesting that Ca_v_ channels play a pivotal role in the PJC-induced vasodilator response. In addition to this, the compound(s) present in F1 could be excellent antagonists of these channels. Concerning this, studies of eucalyptus (*Eucalyptus*) and roselle flowers (*H. sabdariffa*) have demonstrated the contents of several bioactive compounds with vasoactive effects and similar results to those in this study. For example, eucalyptus contains 1,8-cineole (3 mM), which produces a maximum inhibition of 26.7 ± 5.9% for the Ba^2+^ currents through the Ca_v_ channels in the tracheal myocytes of normotensive male Wistar rats [31]. In contrast, an aqueous extract of *H. sabdariffa* (5 mg/mL) induced a maximum reduction of 24.4% for the Ca_v_ channel currents in isolated ventricular cardiomyocytes from normotensive male Wistar rats [28].

After verifying the vasorelaxant effect of PJC and F1 in ex vivo studies on normotensive rats, the next step was to evaluate the vasodilator effect in hypertensive rats. It was established that the administration of a single dose (400 mg/Kg) of PJC in SHRs induced a reduction in both systolic (<120 mmHg) and diastolic (~90 mmHg) BP compared to hypertensive rats that received water as a vehicle (>140 mmHg and >100 mmHg, respectively) 24 h after receiving treatment. Notably, this reduction lasted for 48 h, compared to the 24 h duration of a single dose of Nph, indicating a long half-life and stronger binding with the molecular target. This could provide a more stable antagonist for BP regulation. Similar results have been shown in other studies, where 0.06 mg/day Nph for five days induced ~28% relaxation in hypertensive female Wistar rats induced by propranolol [32], compared with a 17% decrease in systolic BP and a 27% decrease in diastolic BP in SHRs managed with 15 to 30 mg/kg Nph p.o. for 27 weeks [33]. However, to attribute the cause of this effect, more in vivo studies must be carried out with SHRs administered with F1, or the responsible compound must be administered once identified and isolated. Therefore, further research is required.

Betalains, phenolic acids, and flavonoids have been previously reported in fruit species of the genus *Stenocereus* [34]. *S. huastecorum* is a species that was recently described, and its chemical constituents and pharmacological properties were reported using an UPLC-ESIMS approach [15]. The chromatograms obtained from these analyses displayed 31 peaks putatively associated with phenolic compounds and 16 peaks for betalains. There are two types of betalains (betacyanins and betaxanthins), and both types were identified in *S. huastecorum*, with betacyanins (betaine, phyllocactin, and isophyllocactin) being more abundant [15]. These results differ from the chemical compositions of other species of *Stenocereus,* where betaxanthins are more abundant than betacyanins [35], and the most common betaxanthins are indicaxanthin and isoindicaxanthin [14].

The present study performed fractionation-guided by vasodilator activity to identify F1 as the main bioactive fraction. To obtain insights into the chemical nature of the specialized metabolites contained in this bioactive fraction, HPLC-PDA profiling accomplished with UV and 1D NMR analyses was carried out. The chromatograms at 530 nm displayed null signals of betacyanins. A comparison of the chromatograms of PJC vs. F1 obtained at 480 and 254 nm showed a decrement in the intensity of the peaks associated with betaxanthins in F1, suggesting that the compound(s) responsible for the vasodilator effect of F1 are distinct from betalains and phenolics (Figure 4 and Figure 5) [14]. These results were supported by the different UV–vis curves obtained for PJC and F1 (Figure 6).

To explore the kind(s) of specialized metabolite(s) contained in F1, a 1D NMR analysis was conducted. The ^1^H and ^13^C NMR spectra of F1 (Supplementary Figures S4 and S5, respectively) displayed a primary set of signals, which could belong to a single compound or to complex mixture of compounds of the same type (e.g., regioisomers, diastereoisomers, etc.). Thus, the signals in the ^1^H NMR spectrum at *δ*_H_ 5.00 (d, *J* = 3.8 Hz) and 4.41 (d, *J* = 8.0 Hz) were suggestive of two anomeric protons, and together with the set of signals between *δ*_H_ 3.02 and 3.88, supported the presence of two monosaccharide units (Supplementary Figure S4) [36]. The signals shown in the ^13^C NMR spectrum of F1 provide additional support for the structural deductions made from the ^1^H NMR spectrum, allowing us to infer the glycosidic nature of the constituents present in F1. Nevertheless, the set of eight signals at *δ*_C_ 93.42–103.70 (Supplementary Figure S5) suggests that F1 could be composed of a complex mixture of disaccharides, dimeric disaccharides, or even tetrasaccharides as possible vasodilator bioactive compounds [37]. Natural products of the glycosidic type require additional efforts concerning their isolation and purification, mainly by recycling preparative HPLC [38], and the determination of their molecular structure is based on chemical degradative methods and HRMS and 1D/2D NMR analyses. Therefore, the isolation and structural elucidation of the constituents of F1 will be part of a further study.

## 5. Conclusions

The results of the present study confirm that the PJC and F1 from *S. huastecorum* exert a significant vasodilatory effect on both conductance and resistance vessels. This effect was demonstrated to be endothelium-independent and K^+^-channel-independent. A direct relationship exists between the vasodilator effect of PJC and F1 and the inhibition of Ca_v_ channels, which allowed us to establish the inhibition of the Ca_v_ channels as one of the mechanisms by which PJC exerts a vasodilator effect. In vivo studies showed that administering a single dose of PJC to hypertensive rats induced a significant and sustained reduction in BP. Interestingly, this reduction was maintained for up to 48 hours, suggesting promising therapeutic potential for managing AHT.

Bioactivity-guided fractionation studies identified F1 as the primary contributor to this effect. Preliminary chemical characterization of F1 suggested that the responsible compounds are glycosidic in nature, and could be a complex mixture of disaccharides, dimeric disaccharides, or even tetrasaccharides, since there was the presence of two monosaccharide units. However, as the methodologies employed thus far have not been able to precisely identify and quantify the compounds responsible for the antihypertensive effect, it will be crucial to conduct additional studies using preparative HPLC-RI(low- and high-resolution MS data acquisition with the ESI-MS technique and fragmentation studies using MS/MS or MSn experiments) and 2D NMR experiments to identify and isolate the active molecules responsible for this effect and confirm their efficacy and safety in preclinical and clinical models.

## 6. Study Limitations

Additionally, it is important to note the limitations of this study. First, pitaya (*S. huastecorum*) is a seasonal fruit only available in Mexico’s arid lands. Thus, its cultivation, transportation, and consumption can be challenging. Second, to accurately describe the molecule responsible for the vasoactive effect, it is imperative to prove it in different models and determine if synthetic production is possible. It is crucial to conduct additional studies. Furthermore, it is important to determine if other cactus fruits have the same effect but with different seasonality, which would provide a choice of functional fruits throughout the year. Moreover, a proper pharmacologic study should be performed to determine the doses and distribution of this molecule.

## Data Availability

The original contributions presented in the study are included in the article/Supplementary Material. Further inquiries can be directed to the corresponding author.

## Supplementary Materials

The following supporting information can be downloaded at: https://www.mdpi.com/article/10.3390/foods13162631/s1, Figure S1: Histological sections stained with hematoxylin-eosin; Figure S2: Vasoactivity analyses of the pitaya juice concentrate-fraction 1 (F1); Figure S3: Pilot test of spontaneously hypertensive rats (SHR) administered pitaya juice concentrate (PJC); Figure S4: ^1^H NMR spectrum for F1 acquired at 700 MHz in D_2_O/TFA-*d* 99.5:0; Figure S5: ^13^C NMR spectrum for F1 acquired at 175 MHz in D_2_O/TFA-*d* 99.5:0.

## Abbreviations

AHT, arterial hypertension; BP, blood pressure; Cav, voltage-dependent calcium; E+, endothelium presence; E-, endothelium absent; F1, fraction 1; NO, nitric oxide; Nph, nifedipine; PJC, pitaya juice concentrate; SHRs, spontaneously hypertensive rats; TEA, tetraethylammonium; V, vehicle; VSMCs, vascular smooth muscle cells; WKY, Wistar Kyoto.
