# Supplementary material for: Vasorelaxant Effect and Blood Pressure Reduction Potential of Pitaya Juice Concentrate (Stenocereus huastecorum) Associated with Calcium Channel Blockade
Source: Foods. 2024 Aug 22;13(16):2631. doi: 10.3390/foods13162631 (PMC11353776; doi:10.3390/foods13162631)
Supplement: Supplementary file 1 [file foods-13-02631-s001.zip › foods-3100234-supplementary.pdf]

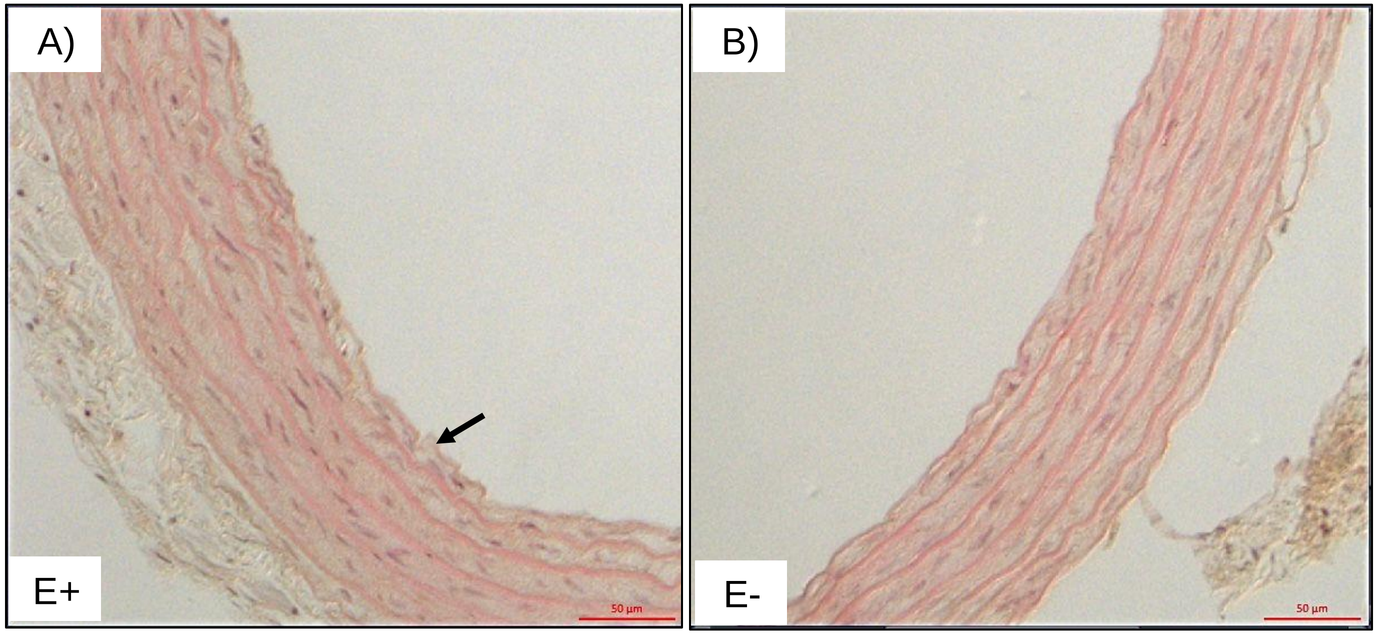

Supplementary Figure S1. Histological sections stained with hematoxylin-eosin. Representative images of (A) aortic ring with endothelium (E +,➔) and (B) aortic ring without endothelium (E-).

FIGURA SUPLEMENTARIA

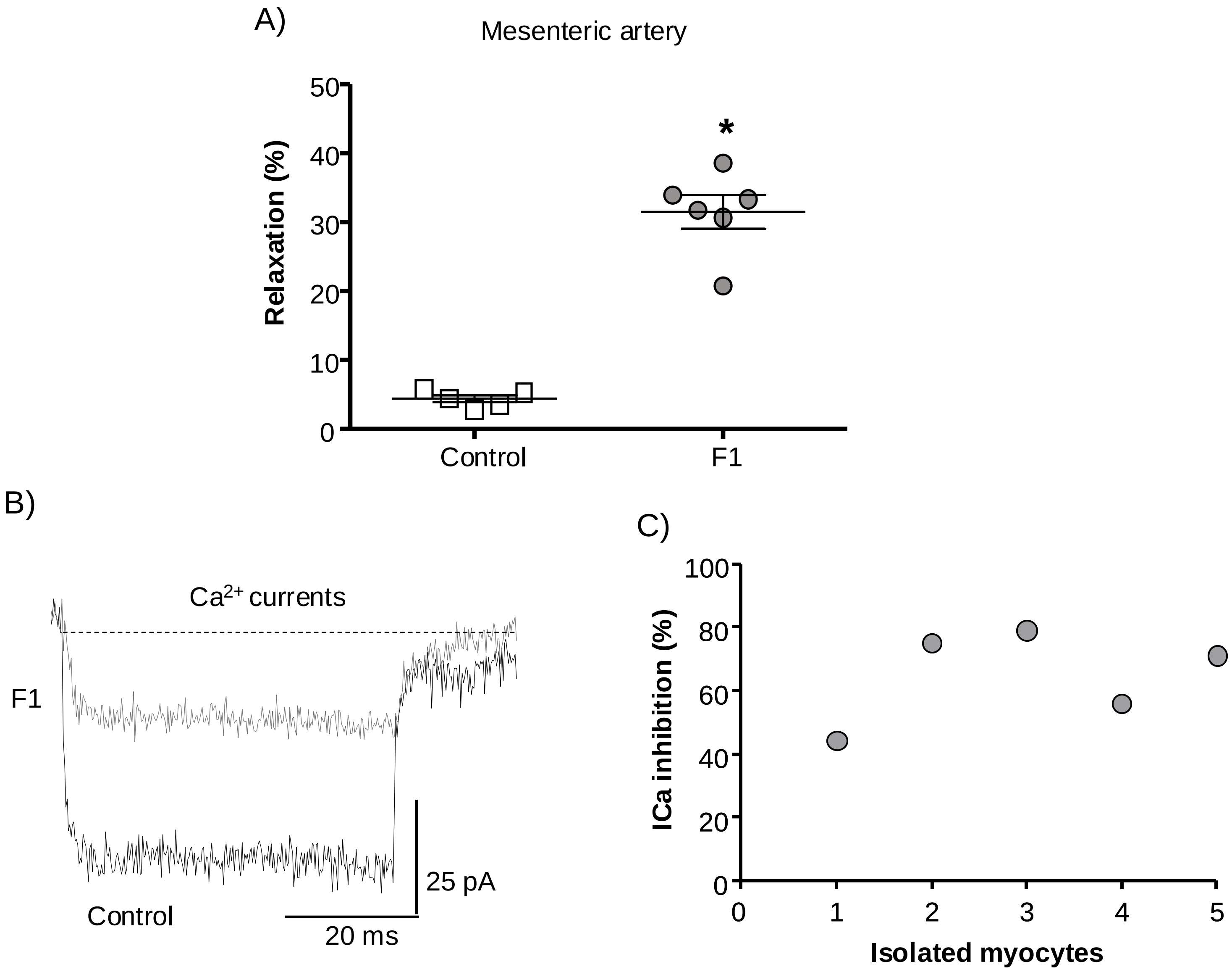

Supplementary Figure S2. Vasoactivity analysis of pitaya juice concentrate (PJC) fraction 1 (F1). (A) Percent relaxation of 5 mg/mL F1 on isolated rings of mesenteric artery previously contracted with phenylephrine (Phe, 10 µM, n=6). (B) Representative traces of Ba^{2+} currents through the voltage-dependent Ca^{2+} channel of mesenteric myocytes recorded at +20 mV from a holding potential of -90 mV in the absence (control) and presence of F1 (20 mg/mL, n=5). (C) Percentage inhibition of Ca^{2+} currents (ICa) with F1 (20 mg/mL). Relaxation was calculated as a percentage of the initial contraction. Data are means ± SEM. * $p < 0.05$ vs. Nph. Millivolts (mV), Picoamperes (pA), Milliseconds (ms).

FIGURA SUPLEMENTARIA

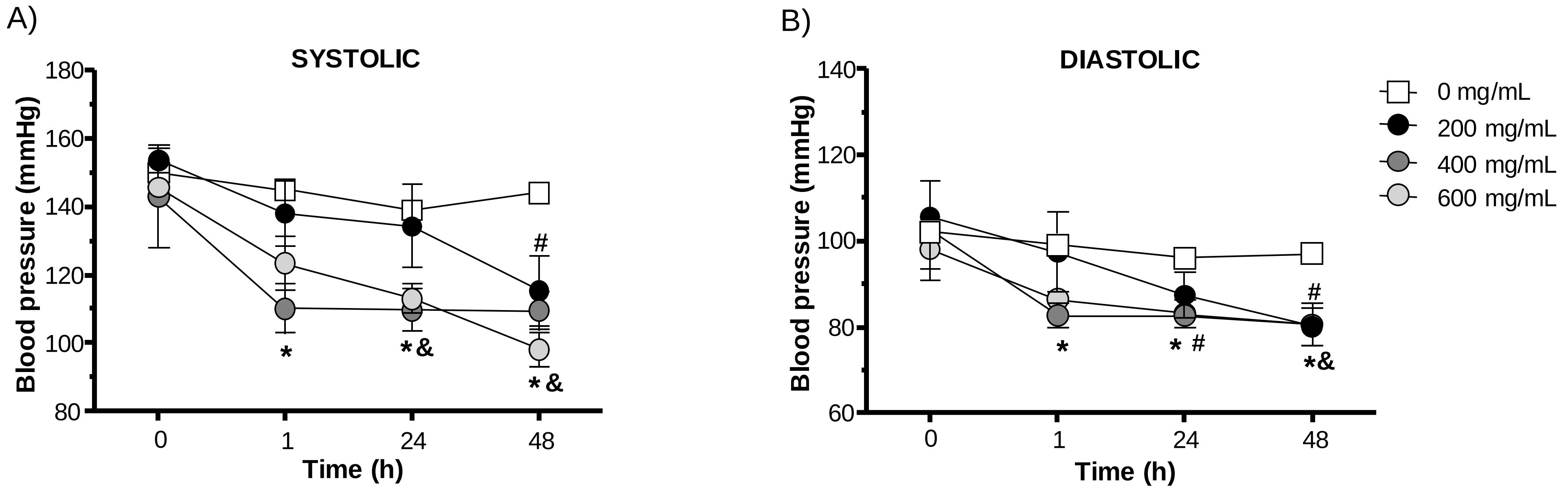

Supplementary Figure S3. Pilot test of spontaneously hypertensive rats (SHR) administered with pitaya juice concentrate (PJC). A) Systolic blood pressure (B) Diastolic blood pressure. Blood pressure recording (mmHg) in SHR administered with increasing doses of PJC (0, 200, 400, 600 mg/mL). Recordings were made at 0, 1, 24 and 48 h, and the data are expressed as means ± SEM (n=3).
# $p<0.05$ *vs.* 200 mg/ml 0 h, * $p<0.05$ *vs.* 400 mg/ml 0 h, & $p<0.05$ *vs.* 600 mg/ml 0 h.

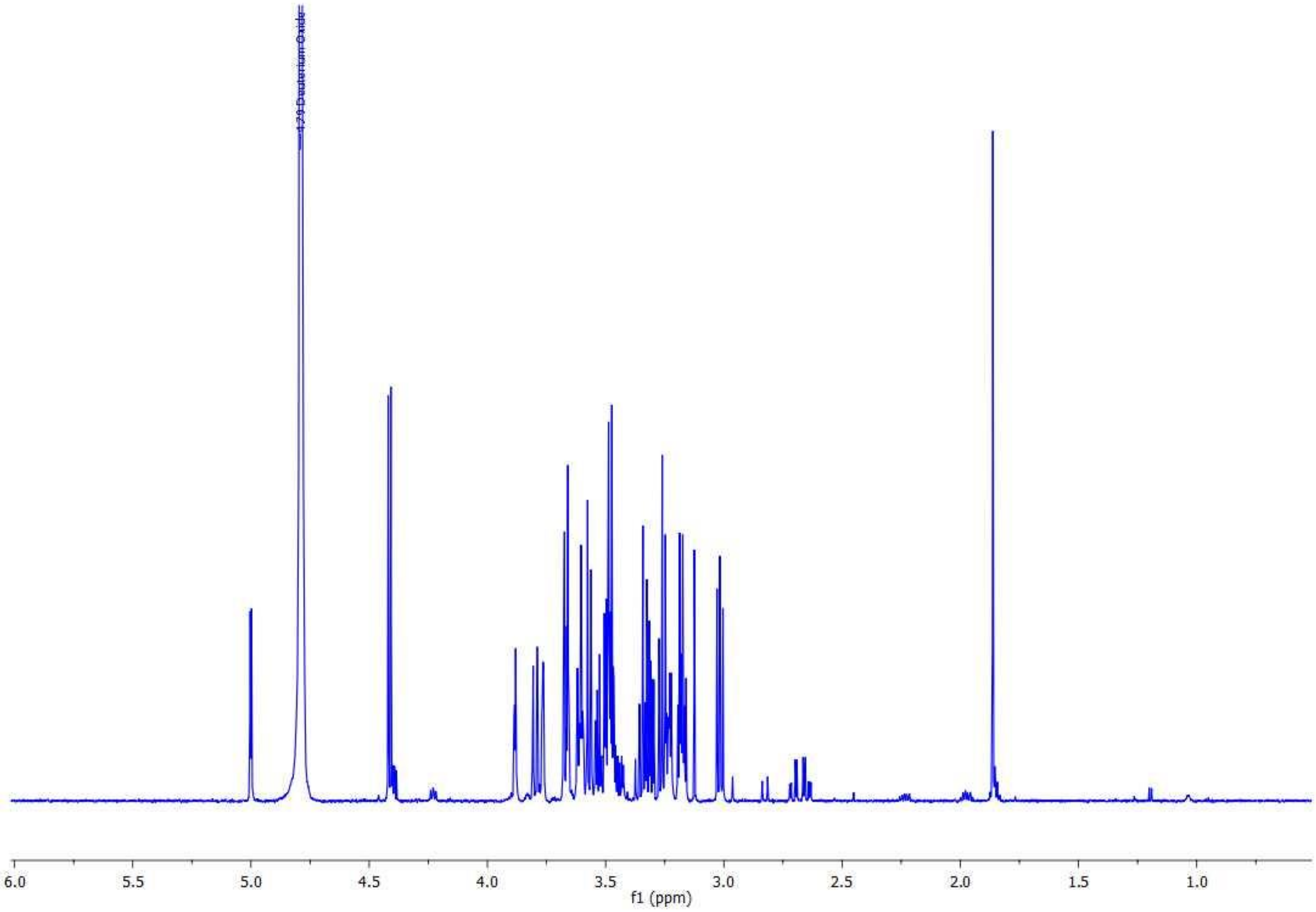

Supplementary Figure S4. [1] H NMR spectrum for F1 acquired at 700 MHz in D_2O/TFA-d 99.5:0

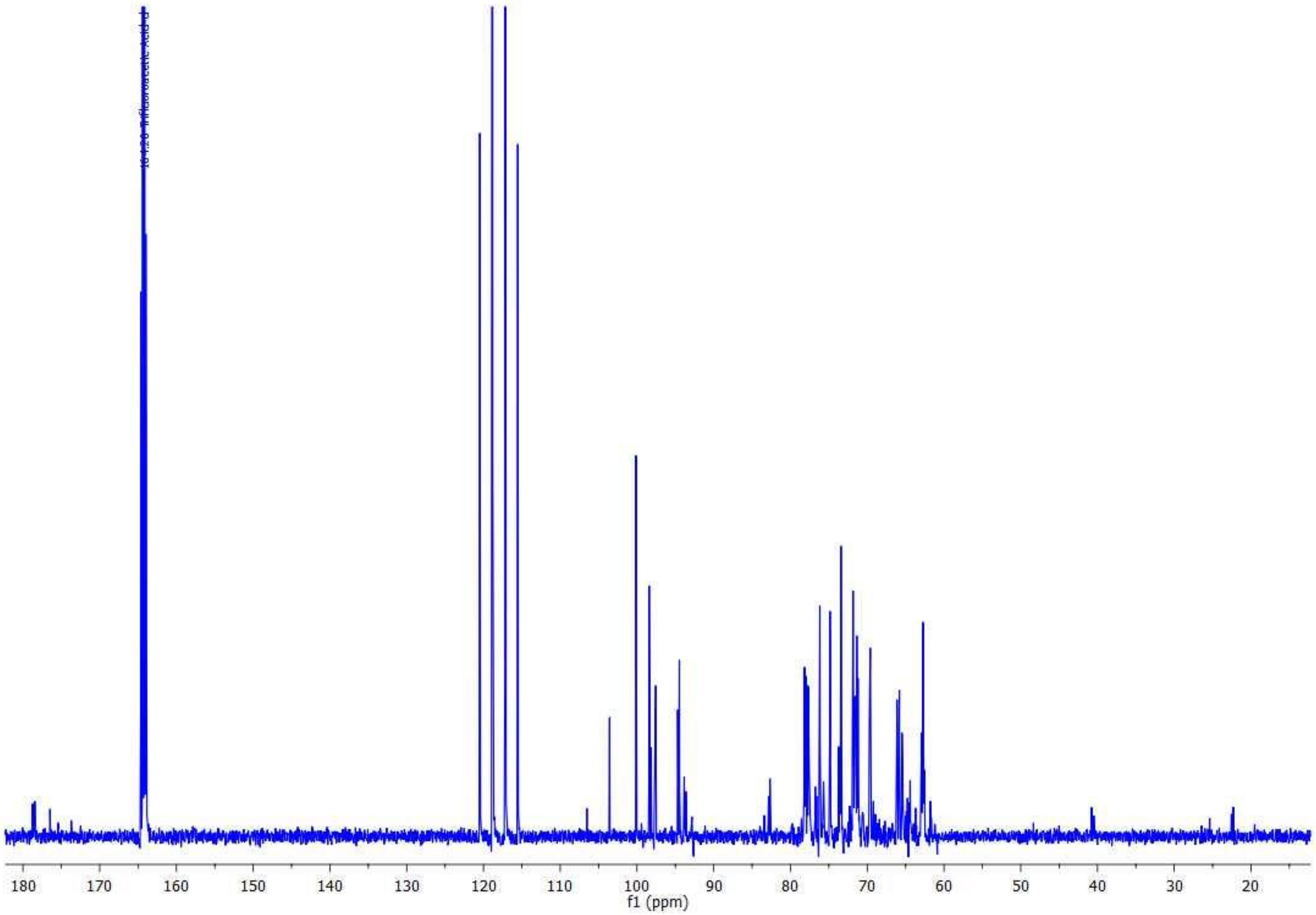

Supplementary Figure S5. ^{13}C NMR spectrum for F1 acquired at 175 MHz in D_2O/TFA-d 99.5:0.5
